# Developmental plasticity and the evolution of parasitism in an unusual nematode, *Parastrongyloides trichosuri*

**DOI:** 10.1186/2041-9139-3-1

**Published:** 2012-01-03

**Authors:** Susan J Stasiuk, Maxwell J Scott, Warwick N Grant

**Affiliations:** 1 AgResearch Limited, Hopkirk Research Institute, Private Bag 11008, Palmerston North, New Zealand; 2 University of Calgary, Department of Comparative Biology and Experimental Medicine, Calgary, T2N 4N1 Alberta, Canada; 3 North Carolina State University, Department of Genetics, Campus Box 7614 Raleigh, 27695-7614, USA; 4 La Trobe University, Genetics Department, Bundoora, 3086 Victoria, Australia

## Abstract

**Background:**

Parasitism is an important life history strategy in many metazoan taxa. This is particularly true of the Phylum Nematoda, in which parasitism has evolved independently at least nine times. The apparent ease with which parasitism has evolved amongst nematodes may, in part, be due to a feature of nematode development acting as a pre-adaptation for the transition from a free-living to a parasitic life history. One candidate pre-adaptive feature for evolution in terrestrial nematodes is the dauer larva, a developmentally arrested morph formed in response to environmental signals.

**Results:**

We investigated the role of dauer development in the nematode, *Parastrongyloides trichosuri*, which has retained a complete free-living life cycle in addition to a life cycle as a mammalian gastrointestinal parasite. We show that the developmental switch between these life histories is sensitive to the same environmental cues as dauer arrest in free-living nematodes, including sensitivity to a chemical cue produced by the free-living stages. Furthermore, we show that genetic variation for the sensitivity of the cue(s) exists in natural populations of *P. trichosuri*, such that we derived inbred lines that were largely insensitive to the cue and other lines that were supersensitive to the cue.

**Conclusions:**

For this parasitic clade, and perhaps more widely in the phylum, the evolution of parasitism co-opted the dauer switch of a free-living ancestor. This lends direct support to the hypothesis that the switch to developmental arrest in the dauer larva acted as a pre-adaptation for the evolution of parasitism, and suggests that the sensory transduction machinery downstream of the cue may have been similarly co-opted and modified.

## Background

One of the hallmarks of the phylum Nematoda is the repeated evolution of parasitism. This important life history strategy has arisen at least nine times in the phylum, based on molecular phylogenies [1-3], to give rise to at least six groups of animal parasites and three groups of plant parasites, each of which are interspersed with non-parasitic relatives. It would appear unlikely that one mechanism of evolution resulted in these multiple events. Our focus has been on the evolution of parasitism in terrestrial nematodes that have a developmentally arrested dauer stage, which evolved in the ancestral mono-phylum Secernentea [4].

These nematodes' life cycles are somewhat diverse, but are all essentially variations on a conserved life cycle composed of four larval stages punctuated by molts and culminating in a reproductive adult. Although nematode parasites have evolved several strategies for infecting their hosts, a common feature of all nematode parasite life cycles is the existence of a developmentally arrested infective stage. For those parasites with simple direct life cycles, the infective stage occurs at the transition point between the outside environment and the host. For those parasites with more complex life cycles, there are multiple arrested stages between the intermediate and definitive hosts [5]. This infective stage of directly developing parasites is non-feeding and so must subsist on internal food stores. Further, the infective larva must also be able to cope with environmental stresses (such as starvation or desiccation) until it encounters a host. A further common feature of parasite life cycles is that the resumption of development that occurs following infection, or transition between hosts, usually requires that the nematode molts. This is observed most clearly in those parasites with simple direct life cycles, in which the infective stage is an arrested third stage larva contained within a highly modified second stage cuticle or sheath. This sheath is shed upon ingestion of the infective larva.

These observations have led to speculation that some feature or features of the generic free-living nematode life cycle may have facilitated the repeated evolution of parasitism in this phylum [4,6-9]. Most particularly, the dauer larva has been suggested as the likely pre-adaptation of free-living nematodes which facilitated the repeated evolution of parasitism [4,6-9]. In free-living nematodes, the dauer larva is a facultatively arrested and stress resistant survival stage, formed in response to environmental cues. The dauer stage is formed in response to high populations and inadequate food resources or suboptimal temperature [10]. More direct evidence for the dauer stage as a precursor to parasitism has come from recent work [11,12], which investigated the regulation of a specific phoretic relationship between the free-living nematode *Pristionchus pacificus *and scarab beetles, and from the investigation of the regulation of development in a nematode parasite of rats, *Strongyloides ratti *[13].

The molecular regulation of dauer formation in *Caenorhabditis elegans*, a free-living nematode, is well understood [14,15]. Components of the key signal transduction pathways have been shown to be present in several other free-living and parasitic nematode species [12,16-19]. Natural selection, however, requires phenotypic variation, and at least some component of that phenotypic variation must be heritable for evolution to occur [20]. This raises the questions: which component or components of the regulation of dauer development show natural genetic variation and are these components likely candidates in a search for a mechanism for the evolution of nematode parasitism? Recent data from wild isolates of *C. elegans *have shown that natural variation in the propensity to form dauer larvae in response to environmental signals does exist [21,22], but that the genetic components of this variation are unlikely to include the core components of the signal transduction pathways discovered by mutagenesis and molecular analysis in the laboratory [12,21,23]. Furthermore, investigation of the function of these signal transduction pathways in a handful of parasitic species suggests that, while some characteristics are conserved (for example, the likely role of dafachronic acid and/or nuclear hormone receptor signaling as the terminal, downstream step; [12]), others vary significantly from *C. elegans *(for example, transforming growth factor beta (TGF-β) signaling; [24]). In addition, comparisons of the transcriptomes of *C. elegans *dauers and the infective larvae of several parasites showed more differences than similarities [25-27], implying that the transcriptional readout of these signaling pathways has diverged significantly between parasites and free-living nematodes. These considerations lead to a conclusion that it is the initial (but as yet undefined) steps of signaling transduction which control the 'dauer switch', in which there is natural variation in *C. elegans*; these steps are likely to be more informative in testing the dauer hypothesis for parasite evolution than the later steps, which have been the subject of much recent research.

**Figure 1:**
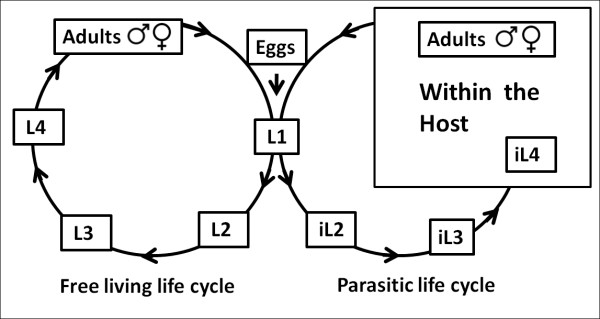
**Life cycle of *P. trichosuri***. *P. trichosuri *first larval stage may develop to either short lived free-living nematodes or into infective larvae (iL3), which require an appropriate host in order to complete their parasitic life cycle. Environmental factors influence this developmental switch.

We describe here an investigation of the characteristics of and variation in parasitic development in an unusual nematode, *Parastrongyloides trichosuri*, in which parasitism appears to be a facultative, developmentally plastic response to environmental cues that are analogous to the environmental cues which determine dauer developmental plasticity in *C. elegans. P. trichosuri *was described initially as an intestinal parasite of the Australian brush tailed possum, *Trichosurus vulpecula *[28]. This species displays an unusual developmental plasticity - at each successive generation a polyphenic switch acts to determine the direction of subsequent development (Figure 1): the switch is activated by environmental signals which the larvae detect. The alternative fates downstream of this switch are direct, uninterrupted development via a free-living (non-parasitic) life cycle to short lived, rhabditiform adults or development via a parasitic life cycle that requires developmental arrest at the infective third larval stage (iL3). Development resumes only when this arrested infective stage infects a host, and culminates in a large and long lived parasitic adult stage in the host's intestinal lumen [28,29]. Thus, in the laboratory at least, *P. trichosuri *can be maintained indefinitely as a free-living nematode by appropriate manipulation of environmental conditions. The parasitic life cycle is thus facultative, and is dependent on the activation of an environment sensitive developmental switch. This species is, therefore, a candidate model in which this switch between free-living and parasitic life cycles may recapitulate one possible trajectory for the evolution of parasitism. In the work described here, our aim was to investigate the likely mechanisms of this unusual developmental plasticity, and hence we focused most particularly on the environmental sensitivity of the switch rather than on the sensory transduction machinery downstream of it. Most importantly, we show that the switch is activated by a chemical cue analogous to the dauer 'pheromone' of free-living nematodes, and that there is genetic variation in the plastic response to this chemical cue. We interpret these observations as directly supporting the dauer hypothesis for the evolution of parasitism, in which the dauer larva of free-living nematodes served as a pre-adaptation that facilitated the repeated evolution of parasitism amongst nematodes. This is, therefore, a system in which evolution has exploited environmentally driven developmental plasticity.

## Results

**Figure 2:**
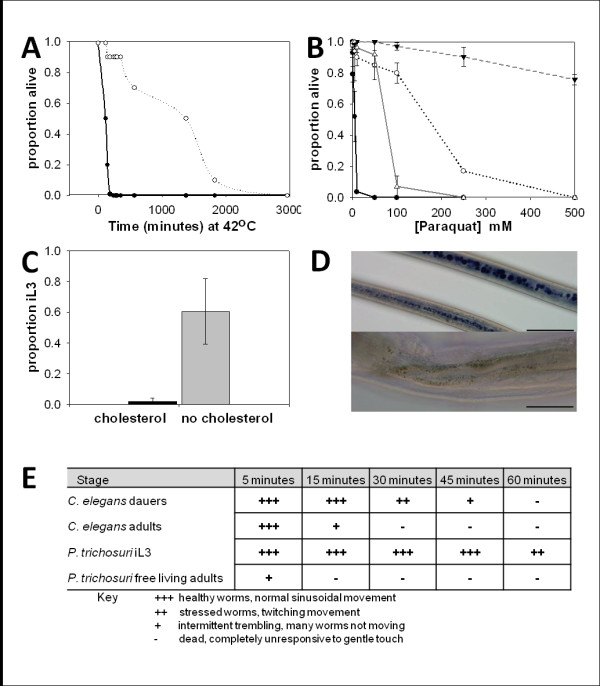
**Biological features of *P. trichosuri *infective larvae**. **(A) **Stress response of *P. trichosuri *infective larvae (dotted line, open circles) and free-living adult stages (black line, closed circles) to incubation (at 42°C) n = 220. **(B) **Resistance to chemical stress (paraquat) of *P. trichosuri *infective larvae n = 1373 (dotted line, open circles); free-living adults (black line, closed circles) n = 257; *C. elegans *dauer (dashed line, closed triangle) n = 1176 and *C. elegans *adults (grey line, open triangle) n = 534. Error bars are standard deviation. **(C) **The effect of cholesterol on *P. trichosuri *iL3 development, n = 311. Error bars are standard deviation. **(D) **Sudan Black staining of two *P. trichosuri *iL3 (top panel) and free-living adults (bottom panel) (scale bar 50 μm). **(E) **Resistance to chemical stresses: *C. elegans *dauer larvae and *P. trichosuri *iL3 larvae resistance to 1% SDS, n = 200.

The original formulation of the dauer hypothesis was supported by the apparent analogy between the biology of dauer larvae of free-living nematodes and the infective larvae of parasitic nematodes. In order to extend the biological analogy between parasites and free-living dauers, *P. trichosuri *free-living adults and infective larvae (iL3) were exposed to various chemical and environmental stresses to determine if the infective larval stage displays the same stress resistance characterized by the dauer stage of *C. elegans*. As shown in Figure 2A, P. *trichosuri *iL3 are better able to withstand elevated incubation temperatures (42°C) compared to the free-living adult stage. In free-living adults of *P. trichosuri*, the time it takes for 50% of individuals to die (LT_50_) when incubated at 42°C is 2 hours, while the LT_50 _of infective larvae is 23 hours. As illustrated in Figure 2B, when *P. trichosuri *infective larvae and free-living adults are exposed for 20 hours to various concentrations of paraquat, a reagent used to test oxidative resistance, the concentration required to kill 50% of a population (LC_50_) of *P. trichosuri *infective larvae is approximately 175 mM paraquat, whereas for the free-living adult stage the LC_50 _is approximately 5 mM paraquat.

The effect of cholesterol depletion on the infective larval development of *P. trichosuri *was also examined. Exogenous cholesterol must be supplied for the reproductive development of *Pristionchus pacificus *and *C. elegans; *when these worms are deprived of cholesterol they will constitutively develop to the dauer stage [12,30]. Cholesterol serves as a precursor for the biosynthesis of Δ4 and Δ7 dafachronic acids (DAs), which are ligands for the nuclear hormone receptor, that is downstream of the signaling transduction pathways controlling the dauer versus reproductive development programs [31,32]. Figure 2C shows the effect on development of culturing *P. trichosuri *larvae on low peptone nematode growth medium (NGM) agar plates for two generations with and without exogenous cholesterol (at 5 μg/mL). There was a significant increase (t-test, t_2 _= 0.05, *P *< 0.001) in the proportion of iL3 development for the F_2 _generation of *P. trichosuri *cultured without exogenous cholesterol.

The *C. elegans *dauer stage is a non-feeding stage of development that relies upon the metabolism of fat stores for energy. In order to determine if the *P. trichosuri *infective larva stage also has increased stores of lipids, *P. trichosuri *free-living adults and infective larvae were stained with Sudan Black, which preferentially stains lipids. Figure 2D illustrates the increased size of Sudan Black-stained lipid stores in infective larvae compared with free-living adults. Lastly, the dauer larvae of *C. elegans *are resistant to environmental insults such as treatment with harsh detergents. As shown in Figure 2E, when incubated in 1% SDS the *P. trichosuri *free-living adults survived less than 15 minutes whilst the infective larval stage was able to withstand exposure to 1% SDS in excess of 1 hour. These data suggest that *P. trichosuri *infective larvae share many of the biological features which characterize the stress resistance of the dauer stage of *C. elegans*.

Entry into the dauer stage in free-living nematodes is mediated primarily by a dose-dependent response to constitutively produced metabolites that have been termed dauer pheromone, or daumone [10,33,34]. We prepared a conditioned medium from *P. trichosuri *cultures using the same method as used to produce the natural dauer pheromone of free-living nematodes [35]. We have previously shown that newly hatched *P. trichosuri *first larval stage (L1) develop into infective larvae in a dose-dependent manner when exposed to conditioned medium [36]. The effect of food availability and temperature on this response to conditioned medium in *P. trichosuri *was also examined.

**Figure 3:**
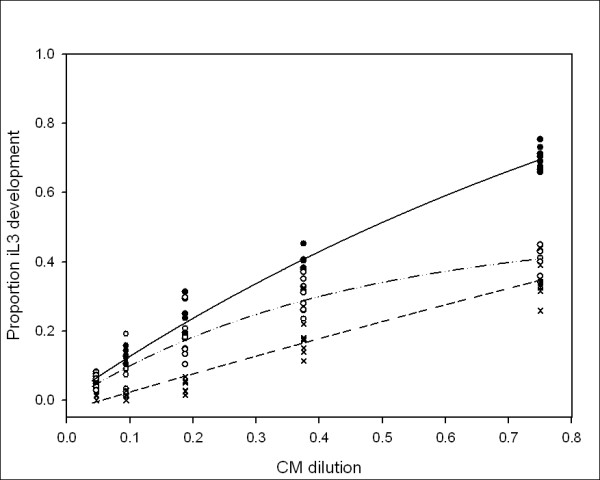
**The effect of various food concentrations on the proportion of *P. trichosuri *iL3 development in response to conditioned medium concentration**. L1 larvae at one individual per microliter were incubated at 20°C in various concentrations of bacterial food source and gentamicin sulfate at 50 μg/μL and serially diluted conditioned medium. Solid line and circle is 0.3% w/v HB101, n = 3249; dashed line and open circle is 0.4% w/v HB101, n = 3174; dotted line and cross is 0.5% w/v HB101, n = 2762. On day 5, the proportion of larvae that had developed into iL3 was scored in six replicate wells. The lower the food concentration available, the more likely the larvae will develop as infective larvae.

Figure 3 shows the effect on infective larval development of newly hatched L1 incubated in a serial dilution of conditioned medium with different concentrations of bacterial *E. coli *HB101 as food source. Culturing in 0.5% (w/v) *E. coli *HB101 resulted in a significantly lower proportion of infective larvae development than culturing larvae in 0.3% (w/v) *E. coli *HB101. The exposure of larvae to conditioned medium at an intermediate food concentration, 0.4% (w/v) *E. coli *HB101, resulted in an intermediate proportion of infective larvae development. The data were analyzed using a non-linear regression. Analysis of variance (ANOVA) suggested that culturing *P. trichosuri *L1 larvae at these different food concentrations resulted in statistically different proportions of infective larvae development (F statistic with pair of degrees of freedom, F_2,125 _= 204.11, *P *< 0.001). Moreover, it was noted from the graphed data, that the shape that this set of reaction norms formed was not a set of parallel lines, but rather a set of diverging lines (F_2,125 _= 7.68, *P *< 0.001).

**Figure 4:**
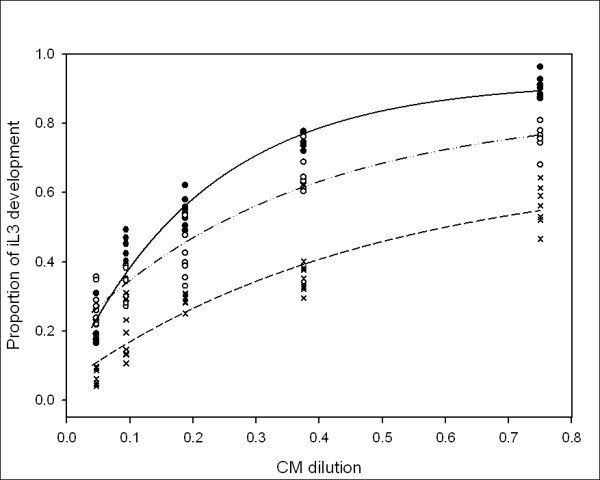
**The effect of various incubation temperatures on the proportion of *P. trichosuri *iL3 development in response to conditioned medium concentration**. *P. trichosuri *L1 larvae at one individual per microliter were incubated at either: 14°C, 20°C or 26.5°C with gentamicin sulfate at 50 μg/μL, and 0.35% HB101 as food source and serially diluted conditioned medium. Solid line and circle is 14°C, n = 5234; dashed line and open is 20°C, n = 7847; dotted line and cross is 26.5°C, n = 7937. On day 5, the proportion of larvae that had developed into iL3 was scored in six replicate wells. The lower the temperature in which the *P. trichosuri *were raised, the more likely they develop as infective larvae.

Figure 4 shows that incubation at 14°C resulted in a higher proportion of *P. trichosuri *development to infective larvae when compared to incubation at 26.5°C. Incubation temperatures of 20°C resulted in an intermediate result except at the lowest dilution, where there was no significant difference between 14°C and 20°C. The data was analyzed using non-linear regression. An ANOVA suggested that culturing *P. trichosuri *at the different incubation temperatures resulted in statistically different proportions of infective larval development (F_2,110 _= 249.74, *P *< 0.001).

These results confirm that there is a factor or factors in the conditioned medium, produced by the worms, which influence their developmental decision and that the response to this factor or factors is influenced both by temperature and by food availability. The modification of the pheromone response by either temperature or food differs, as illustrated by the different shape of the family of curves for the two treatments. The set of dose-response curves for conditioned medium exposure (or reaction norms) for the different temperatures (Figure 4) are parallel curves that differ in their *y-*asymptotes. In contrast, the reaction norms for the different food concentrations (Figure 3) have different slopes, with the low food concentration resulting in a more acute response to the different concentrations of conditioned medium then the higher food concentrations.

In order to analyze how the relationship between the effect of food on the response to conditioned medium and the effect of temperature on the response to conditioned medium differed, the spread (or fan coefficients) for the sets of reaction norms were calculated. The fan coefficients (Table 1) for food are higher than the fan coefficients for temperature, that is, the lowest fan coefficient for the food curves is larger than the highest fan coefficient for the temperature curves. Since there is no overlap in the fan coefficient values, this suggests the food response lines spread more than the temperature response lines. Thus, food availability influences the developmental response of *P. trichosuri *to conditioned medium in a different manner than temperature does, which suggests that a complex relationship exists between environmental factors and the developmental switches they modulate.

**Table 1:** Fan coefficients of reaction norms for differing incubation temperatures and food availability

ψ **for all food curves**	0.05433
fan coefficient φ (for curves 0.3% w/v and 0.4% w/v)	0.1131
fan coefficient φ (for curves 0.3% w/v and 0.5% w/v)	0.0520
fan coefficient φ (for curves 0.4% w/v and 0.5% w/v)	0.1130

ψ **for all temperature curves**	0.003094

fan coefficient φ (for curves 14°C and 20°C)	0.004068
fan coefficient φ (for curves 14°C and 26.5°C)	0.015151
fan coefficient φ (for curves 20°C and 26.5°C)	0.00714

A measure of the shape of the curves is given by the variance of the estimated asymptotes - estimates of M: ψ = variance (asymptotes - minimum fitted values for each curve). The best estimate of spread is ψ for each data set, the bootstrapped values for each pair of curves is φ*. There is no overlap between the two groups therefore the spread for food response is significantly greater than the spread for temperature response.

**Figure 5:**
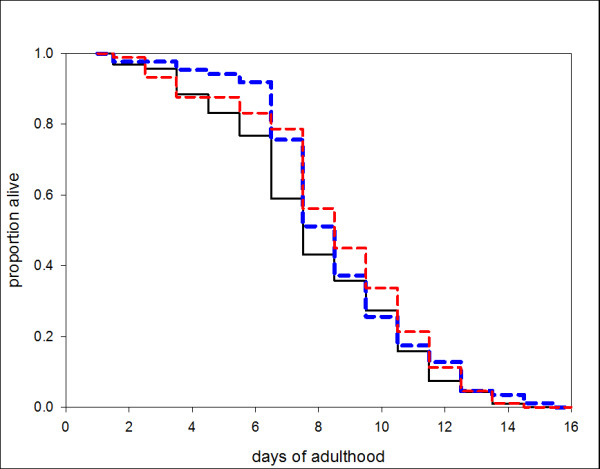
**The lifespan of free-living *P. trichosuri *adults, incubated in various liquid medium**. *P. trichosuri *free-living worms were reared until L4 stage (approximately 36 to 48 hours). At least 85 individual worms were plated in 96-well plates containing liquid nematode growth medium with low peptone, red line, that was either 50% conditioned medium (supplemented with semi-purified conditioned medium to a ratio of 1:10), black line; or in 50% bacterial conditioned media control (BAC), blue line. The worms were cultured at 20°C and observed daily. Worms were scored as dead when they no longer responded to touch. There was no significant difference in lifespan between the various culture media.

The insulin/insulin-like growth factor (IGF) signaling pathway has been shown to influence lifespan in *C. elegans, Drosophila melanogaster *and possibly mammals [37-41]. In *C. elegans *biology, insulin signaling controls both dauer development and lifespan, and it has been shown by Kawano *et al*. [42] that *C. elegans *natural pheromone acts pleiotropically to influence dauer development at early larval stages and to extend lifespan when worms are exposed at later stages of development. In order to determine whether the *P. trichosuri *conditioned medium is able to influence lifespan as well as influence infective larval development, *P. trichosuri *at a late larval stage (the fourth larval or early adult) were exposed to either conditioned medium or to the bacterial control medium (BAC). The results shown in Figure 5 indicate that there was no extension in lifespan. The concentrations of conditioned medium used in the lifespan extension experiments were at concentrations that can induce the development of approximately 80% to 90% infective larvae.

**Figure 6:**
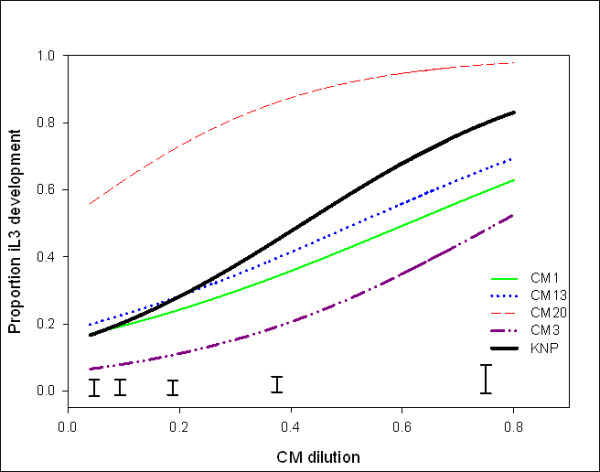
**Infective larvae development of *P. trichosuri *inbred lines versus conditioned medium**. Inbred lines were created as described in the Methods section. To determine developmental reaction norm of these inbred lines, a conditioned medium bioassay was performed. Each inbred line was tested in two possums, on three separate days, each with six replicate wells of each dilution of conditioned medium. Results were pooled and graphed, (n > 7,500 for each line). The data was analyzed by logistic regression and the fitted lines shown above. Inbred lines CM20 and CM3 differ significantly at all dilutions of the conditioned medium compared to the outbred line (KNP) (*P *< 0.001). Least significant difference error bars at the bottom of the graph represent the least significant difference (at 5% significance level) between any two inbred lines at the concentrations of conditioned medium tested.

In order to investigate the genetic variation associated with responses to conditioned medium, we created inbred lines of *P. trichosuri *by pre-selection for sensitivity or resistance to conditioned medium signal followed by several generations of brother-sister mating. These lines were assayed for infective larval development in response to the serial dilution of conditioned medium and compared to the outbred parental (KNP) line, as shown in Figure 6. The norm of reaction for inbred line CM20 was significantly greater than the KNP outbred line (F_2,192 _= 715.66, *P *< 0.001). Inbred line CM3's norm of reaction was significantly less than the outbred KNP line (F_2,157 _= 198.29, *P *< 0.001). The slope and intercept parameters from these straight-line regressions were analyzed to compare the five inbred lines using ANOVA. The intercepts of inbred line CM3 and CM20 differed from the outbred KNP line (F_4,11 _= 16.44, *P *< 0.001); line CM3 had a significantly lower intercept then outbred KNP, and the inbred line CM20 had a significantly higher intercept than KNP. The other two lines had average intercepts similar to KNP. An ANOVA did not find any significant differences in slope between the lines (F_2,11 _= 0.525, *P *= 0.84).

## Discussion

Much of the evidence in support of the dauer hypothesis for the evolution of parasitism is indirect. We have shown here, and elsewhere, that *P. trichosuri *may offer a model in which this hypothesis can be tested more directly [36]. We have established that the infective larvae of *P. trichosuri*, in common with those of many other parasites that have been examined, share a number of stress resistance characteristics with dauer larvae. Our results show that *P. trichosuri *infective larvae share several important biological characteristics with the *C. elegans *dauer stage, such as resistance to chemical and environmental stress. First, *P. trichosuri *infective larvae were able to withstand incubation at temperatures of 42°C for ten times longer than the adult stage, whilst the *C. elegans *dauer stage was able to survive three times longer than the adult stage at an elevated incubation temperature of 37°C [43]. Second, the ability to withstand incubation in 1% SDS is a defining characteristic of the *C. elegans *dauer stage [44,45]; *P. trichosuri *infective larvae showed a similar ability to survive longer in SDS compared with free-living adults. Third, both *P. trichosuri *infective larvae and the *C. elegans *dauer stage survived longer in the oxidative chemical stressor, paraquat, compared to adult worms. Fourth, newly formed *P. trichosuri *infective larvae had large lipid stores, suggestive of the importance of lipids for metabolism during the infective stage of development. The *C. elegans *dauer stage relies on lipid stores and increased gluconeogenesis for metabolism [43,46]. The infective larvae of *Ancylostoma tubaeforme, A. caninum *and *Uncinaria stenocephala *have also been shown to rely on lipid stores for metabolism [47,48].

This work has also shown that exogenous cholesterol is required for the free-living development program of *P. trichosuri*. Exogenous cholesterol is required for *C. elegans *reproductive development as they are unable to synthesize steroids *de novo *[30]. The cholesterol serves as a precursor for the production of DAs, which act as ligands for DAF-12 (a nuclear hormone receptor) [32]. Similarly, it has been demonstrated that *S. papillosus *also required the addition of exogenous cholesterol for free-living development, as the depletion of cholesterol resulted in 100% infective larval development in this species [12]. Consistent with the effects of cholesterol depletion, the application of DAs reduced infective larval formation in *S. papillosus *and *S. stercoralis *in favor of the free-living morph [12,49]. It was also demonstrated that these DAs bind to and activate orthologs of the nuclear hormone receptor DAF-12 in *S. stercoralis *and the hookworms *A. caninum, A. ceylanicum *and *Necator americanus *[49].

We have shown previously [36] that *P. trichosuri *larvae develop to infective larvae in response to a biological factor or factors found in conditioned medium in a dose-dependent manner reminiscent of the population indicator, dauer pheromone, of *C. elegans *[10,50]. In this report, we explore the nature of the pheromone response and its interaction with other environmental signals in greater detail.

In *C. elegans*, the pheromone signal is transduced through the insulin/IGF and TGF-β signaling pathways, which converge to regulate both DAF-12 and the production of its ligands, the Δ4 and Δ7 DAs [14,30]. In addition to this dauer signal transduction role, the insulin/IGF pathway strongly influences lifespan in *C. elegans *[51]. However, there are contradictory reports in the literature [42,52] as to whether pheromone exposure alone is sufficient to extend adult lifespan in *C. elegans*, hence we wanted to determine if the biological factor or factors contained in the *P. trichosuri *conditioned medium had an effect on the lifespan of free-living *P. trichosuri *adults. Conditioned medium at a concentration that induces approximately 80% to 90% infective larvae was applied to late stage larvae of *P. trichosuri*, which are already fully committed to free-living development, and the adult lifespan of these worms was measured. We failed to detect any effect of *P. trichosuri *conditioned medium on free-living adult lifespan, in agreement with [52], who also observed no effect of *C. elegans *pheromone on the lifespan in that species. Thus the lifespan of the free-living adults does not appear to be extended by the pheromone alone. Food availability and temperature both play a role in *P. trichosuri *infective larval development: lower incubation temperatures and lower food concentrations both resulted in a greater proportion of infective larvae at a given concentration of conditioned medium. One way to quantify the relationship between genotype, the environment and phenotype is by describing 'norms of reaction' of an organism by plotting the phenotype of an organism against a range of possible environments. This was done by measuring the polyphenic developmental choice between the free-living morph and infective larval morph in response to a range of environmental signals. We found that *P. trichosuri *larvae were able to integrate multiple environmental signals in order to make their developmental decisions. The reaction norms of response to conditioned medium at different temperatures gave the same shaped curves with different asymptotes, with low temperatures resulting in greater infective larval development. The *y*-intercept can be interpreted as a default setting for the developmental decision, that is, some L1s will develop to iL3 even in the absence of a signal. Thus, temperature acts to modify the default setting for conditioned medium response, but not the degree of responsiveness. This phenomenon is also observed in the response of *C. elegans *to natural pheromone, and has a genetic component [21]. In contrast, the response to conditioned medium under conditions of different food availability resulted in reaction norms with similar *y*-intercepts but with different slopes: *P. trichosuri *responded more strongly to conditioned medium under conditions of low food availability, as illustrated by the steeper slope of the reaction curve at the lowest food availability. This implies that low food availability does not change the default setting of the switch between morphs but rather makes the larvae more sensitive to the conditioned medium signal. By analogy with the pheromone of *C. elegans, P. trichosuri *conditioned medium is most likely a measure of population density. There is, therefore, an obvious adaptive advantage for worms to be more sensitive to increases in population density when food concentration is low rather than high.

Nutrition also effects the homogonic or heterogonic development of the parasitic nematodes *S. planiceps *[53] and *S. ransomi *[54]. In these species, either a lower concentration of food or food that has depleted nutritional value due to washing and autoclaving results in a higher proportion of infective larval development. With respect to temperature, it has been found that extremes in culturing conditions (either high or low incubation temperatures) for *S. fuelleborni *result in a slight increase in homogonic development [55] and that a lower temperature also results in more infective larval development in *S. ratti *[13,56,57], *S. planiceps *[58] and *S. papillosus *[59]. However, the lower incubation temperatures used in the *Strongyloides *experiments may have had an effect on the growth of bacteria used as the food source, and as such the environmental signal tested may have been as much food availability as temperature. In contrast, it is higher temperatures that result in the formation of more dauers or infective larvae in *C. elegans *and *S. stercoralis*, respectively [10,60,61]. The differences in the developmental response to temperature may be an indication of a response calibrated against the temperatures at which each species ceases development, that is, finds stressful. In *C. elegans*, this threshold is around 27°C, whereas for *P. trichosuri*, the upper limit for development is in excess of 30°C (Grant, unpublished). While the upper temperature boundary for the *Strongyloides *species is not known, it may be relevant that *S. planiceps *and *S. papillosus *are found in equatorial regions and may find cooler temperatures stressful [61]. A food signal inhibits dauer formation in *C. elegans *[10].

The developmental plasticity displayed by these parasites (in *Strongyloides *species in the first generation only, and in *Parastrongyloides *species at each generation), allows the worms to alter their development according to a set of environmental cues. When the environmental cues suggest there is likelihood of successfully reaching reproductive adulthood as a free-living nematode, development proceeds directly to the free-living morph, and this results in an expansion of the population. For example, Grant *et al*. have shown that successive free-living generations in fecal cultures of *P. trichosuri *can expand the population of iL3 produced by several orders of magnitude over the starting population of eggs in the feces [36]. Alternatively, when environmental cues such as increased competition for resources or non-optimal temperatures signal to an L1 that it is in a stressful environment with a decreased likelihood of reproducing successfully as a free-living worm, it enters diapause as an infective larva in the hope of eventually encountering a host to complete its life cycle. The evolution of a developmentally plastic life history strategy that permits the choice between a short-lived, short generation time morph with very high reproductive potential (the free-living life cycle), and a long-lived, longer generation time morph (the parasitic life cycle) can be viewed as an evolutionary advance on the life history strategy of free-living and necromenic nematodes such as *C. elegans *and *P. pacificus*. The free-living morph serves to expand the population, thereby maximizing the chances that at least some iL3 will make it through the population bottleneck that transmission to a host imposes. Parasite life history strategies that include a proliferative phase that increases transmission potential are common in other taxa (for example, trematodes and cestodes), but relatively rare amongst nematode parasites.

The hypothesis that dauer larvae are a pre-adaptation for the evolution of parasitism implies that at least some of the steps of that evolution should involve changes in the regulation of dauer biology. Natural selection requires that there be phenotypic variation, and that at least a portion of that phenotypic variation be heritable. Harvey *et al*. have shown that genetic variation in pheromone response exists between wild isolates of *C. elegans *[22], so we sought evidence for genetic variation in conditioned medium response in *P. trichosuri *using a similar approach, which combined selection for high or low response with inbreeding by repeated single pair brother-sister matings. Lines CM3 and CM20 differed from each other, and from the outbred parental line, by several standard deviations in their conditioned medium response at all concentrations tested, while lines CM1 and CM13 were similar to each other and to the parent despite several generations of inbreeding. This genetic variation in responsiveness is a good evolutionary bet-hedging strategy which ensures that, under most environmental conditions, some proportion of the population will arrest as infective larvae and some will continue to reproduce. For example, even at the highest concentration of conditioned medium it was not possible to achieve 100% infective larval development, suggesting that there is a reservoir of worms within a population that do not respond easily to this signal and develop as reproductive free-living adults. This is particularly important when one considers that, for most parasites, only a small proportion of infective larvae are likely to encounter a host and most will succumb to starvation or other environmental stressors before they do so [62]. Consequently, this variation facilitates a gradual transition from a predominantly reproductive free-living population to a predominantly arrested, non-reproductive one (waiting for a host) and thus may maximize the reproductive fitness of the population. It is also consistent with the hypothesis that selection on dauer biology may have occurred as a component of the evolution of parasitism.

The creation of the inbred lines described here, which demonstrates that there is a genetic component to the switch between morphs, paves the way for genetic analysis of this key life history trait in a parasite. The nature of genetic differences between these inbred lines is at present unknown. The differences may be in the way the worms detect the biological signaling factor or the way in which this signal is transduced or even the expression levels of the remodeling or developmental genes involved in infective larvae development. Classical quantitative trait locus analysis and gene expression analysis of these inbred lines may elucidate some of these issues.

## Conclusions

The developmentally arrested and stress resistant dauer stage of free-living nematodes shares much biology with the infective larvae stage of parasitic nematodes; the cues that influence the developmental switch to infective larvae stage in *P. trichosuri *are the same environmental cues which influence dauer development in free-living nematodes (temperature, food, and population density sensed through a pheromone-like biological factor). This suggests that the dauer stage may have served as a pre-adaption for the development of the infective larvae stage in the evolution of parasitism. The sensitivity to the pheromone-like biological factor may have served as a candidate for a mechanism of evolution of nematode parasitism; we have shown that there is phenotypic variation within a population of the sensitivity to this environmental cue, and that this sensitivity is heritable, making it a candidate upon which natural selection may act.

The facultative developmental switch at each successive generation allows *P. trichosuri *to serve as a candidate model with which the dauer hypothesis may be tested. The short generation time allows for ease of maintenance and experimental setup. The large brood sizes allows for robust statistical analysis. The facultative free-living life cycle allows for ease of maintenance within a laboratory setting and classical genetic analysis - a feature missing from many other parasitic nematodes.

## Methods

### Parasitological Procedures

Australian brush tailed possums (*T. vulpecula*) possums were maintained as previously described [36]. Animal Ethics for experiments described was granted by the Wallaceville Animal Ethics Committee AEC#273. *In vitro *cultures of *P. trichosuri *were maintained as previously described [36].

### Assays for environmental stress and lipid staining

To determine resistance to elevated temperature, synchronized *in vitro *cultures of either adult or infective larvae were placed onto low (1/10) peptone NGM agar plates [63] with *E. coli *HB101 and possum fecal material as a food source and then incubated at 42°C. At timed intervals, animals were assessed and scored as dead when they no longer responded to touch. To determine resistance to 1% SDS, either *C. elegans *(adults or dauers) or *P. trichosuri *(free-living adults or infective larvae) were exposed to 1% SDS as previously described [44,45]. To determine resistance to oxidative stress, paraquat (methyl viologen dichloride hydrate) (Sigma-Aldrich, Australia M2254) was dissolved in low peptone liquid NGM and approximately 40 *P. trichosuri *or *C. elegans *of either adult stage or infective/dauer stage were exposed to various concentrations of paraquat. Worms were assayed for motility after 20 hours incubation at 20°C and scored as dead when they no longer responded to touch. To determine lipid stores of *P. trichosuri *infective larvae, worms were stained with Sudan Black, which preferentially stains lipids, as described previously [64]. Worms were visualized on an Olympus, USA BX-UCB microscope.

### Effect of exogenous cholesterol on infective larvae development

*P. trichosuri *adults were cultured on low peptone NGM agar plates either with or without cholesterol (at 5 μg/mL) for two generations; *E. coli *HB101 and possum fecal material were used as food sources. The F_2 _generation larvae were then scored visually for either free-living development or iL3 development.

### Conditioned medium

The procedure for preparation of a *P. trichosuri *conditioned medium was based on the preparation of dauer pheromone for *C. elegans *[50] and as previously described [36]. *P. trichosuri *conditioned medium contains a biological factor or factors which induce development to infective larva in a dose-dependent manner, analogous to the population density indicator *C. elegans *dauer pheromone. A control medium (BAC), was made by the same procedure, but without nematodes to produce the biological factors. The same batch of conditioned medium was used for all developmental assays described herein.

### Effect of temperature and food availability on *P. trichosuri *developmental fate

All the worm bioassays had cholesterol supplied exogenously at 5 μg/mL in the NGM culture medium (cholesterol is a component of NGM). The effect of temperature and food availability on the developmental fate of *P. trichosuri *larvae was assessed. A two-fold serial dilution series of the conditioned medium was performed, which consisted of six replicates of approximately 100 larvae for each of the dilutions. These were performed in liquid low peptone NGM using freshly hatched larvae at one L1 per microliter and 50 μg/mL gentamicin sulfate to inhibit bacterial growth. For the food availability experiment, the *E. coli *HB101 food source was varied (0.3% w/v, 0.4% w/v and 0.5% w/v) and the incubation was held constant at 20°C. For the assessment of temperature effects on development, the *E. coli *HB101 food source was kept constant at 0.35% w/v and the incubation temperature was varied (14°C, 20°C and 26.5°C). On day 5 the proportion of free-living and infective larvae were calculated for each treatment.

### Free-living lifespan of *P. trichosuri*

To estimate the lifespan of free-living worms, synchronous cultures were established in conditions that favored free-living development; then at the L4 or early adulthood stage (approximately 36 hours), individual worms were transferred to a 96-well plate containing low peptone NGM broth and either 50% of conditioned medium or a BAC control with 0.35% w/v *E. coli *HB101 as the food source. Worms were monitored daily for viability and every three days a 2 μL aliquot of 0.5% w/v *E. coli *HB101, suspended in the appropriate media, was added to maintain the availability of a food source. Worms which did not respond to light touch were deemed to be dead. Lifespan was calculated as the time in days from the initiation of the culture to the death of each individual worm.

### Creation of *P. trichosuri *Inbred lines

To investigate whether there may be a genetic component to the variation in sensitivity to conditioned medium concentration in *P. trichosuri*, inbred lines with either high or low sensitivity (compared to the median of the sensitivity of the outbred KNP isolate) were created in a two-step procedure. First, the outbred population was selected over three generations for a high or low response to conditioned medium, and then the divergent populations were used as the starting point for 10 generations each of single pair brother-sister matings. Thus, in order to select for larvae with increased sensitivity to conditioned medium, a *P. trichosuri *outbred population (KNP) was cultured in a low concentration of conditioned medium (20% v/v) which would normally result in < 10% iL3 development. The infective larvae population (G1) that developed (that is, worms that were sensitive to a low concentration of conditioned medium), were subsequently used to infect a possum and eggs were collected from the feces. This next generation was subjected to incubation in 10% (v/v) conditioned medium and the resulting population of infective larvae that developed were used to infect a possum. The next generation (G2) was then subjected to incubation in 5% (v/v) conditioned medium, which would normally induce very little infective larval development, and the resulting population of infective larvae (G3) was used to infect a possum. The free-living progeny from this possum were used as the starting population of 10 generations of single pair brother-sister matings to create an inbred line with high sensitivity to the conditioned medium. In an analogous experiment to select for low levels of response to conditioned medium, *P. trichosuri *were selected for resistance to conditioned medium by incubating freshly hatched L1's in 80% (v/v) conditioned medium for 36 hours. The few worms that failed to develop to infective larvae, that is, remained free-living, served as a starting population for 10 generations of single pair brother-sister matings to create inbred lines with a low conditioned medium response.

The majority of single pairs in the first generation of both inbreeding experiments produced progeny. For each of the selected populations, a further eight single pairs from each well containing progeny (in this case, the pairs were brother and virgin sister) were selected and cultured. This process of single brother-virgin sister mating was continued for 10 generations in parallel for each of the selected populations. During this time, the majority of the lines died out, leaving a small number of inbred lines for each selected population. Lines CM20 and CM1 were created from the larvae selected for high sensitivity to conditioned medium and lines CM3 and CM13 were created from larvae selected for low response or resistance to the conditioned medium signal.

Harvey *et al*. found that, for *S. ratti*, parasites from an immune host have a greater proportion of heterogonic versus homogonic offspring [56]. To reduce the possibility that the divergence in the conditioned medium response of *P. trichosuri *infective larva development between inbred lines was due to the immune status of the possum host, rather than a genetic component of the inbred lines themselves, the conditioned medium response of each inbred line was tested after passage through two different hosts (matched for age, weight, sex and prior infection history) and found not to differ. We concluded, therefore, that the difference in response between lines was independent of the host. Inbred lines were also assayed for infective larvae development in response to serial dilution of the conditioned medium. Each dilution series of conditioned medium had at least five replicates of each dilution and were repeated in three separate trials; the average proportion of iL3 development was graphed.

### Statistical analysis

The data from each dilution series was modeled using logistic regression, where conditioned medium concentration was the explanatory variable and the logit (or logistic transformation) of iL3 proportion was the response. This was a joint analysis of all dilution series together with the inbred line as a grouping factor in the model. The slope and intercept parameters from the above regressions were compared for each inbred line using ANOVA. Analysis for each data set was performed by fitting least squares to each curve.

The set reaction norms for developmental response to temperature, in a serial dilution of conditioned medium, produced a set of seemingly parallel curves. In contrast, the set of reaction norms for developmental response to different food availability, in a serial dilution of conditioned medium, produced a set of curves in which the response was more acute the lower the food availability. In order to analyze the shape of these families of reaction norms, the measure of spread for the two treatments (food availability or temperature) were analyzed as follows: the starting point M of the response variable was subtracted from the asymptote for each set of exponential curves and the variances of the spread for the sets of curves were analyzed. To each set of data, a family of c exponential curves was fitted by least squares. From c variants of fitted values within a family, c minimum fitted values M were estimated and each was subtracted from the asymptote estimate of its respective curve to give c heights (h(1...c)). The variance of the estimates of φ was then estimated:

$$\psi =\sigma^{2}\left(\varphi \left(1\ldots \text{c}\right)\right)\ldots .$$

to get an estimate of the range of ψ for each family of curves (all combinations of c-1 curves per family were used); and a set of re-sampled φ* estimates, (bootstrapped values for a more robust estimate) along with ψ gave an estimate of the range and variance of φ. If the ranges failed to overlap, the spread of each family of curves was considered different.

The t-test was used, assuming a two-tailed distribution with unequal variance to test developmental response in the absence of exogenous cholesterol.

## Abbreviations

ANOVA: analysis of variance; DA: dafachronic acid; IGF: insulin-like growth factor; iL3: infective third larval stage; L1: first larval stage; NGM: nematode growth medium; TGF-β: transforming growth factor beta.

## Competing interests

The authors declare that they have no competing interests.

## Authors' contributions

SJS and WNG conceived and designed the study, all authors wrote the manuscript. SJS collected the data and all authors analyzed the results. All authors read and approved the final manuscript.
